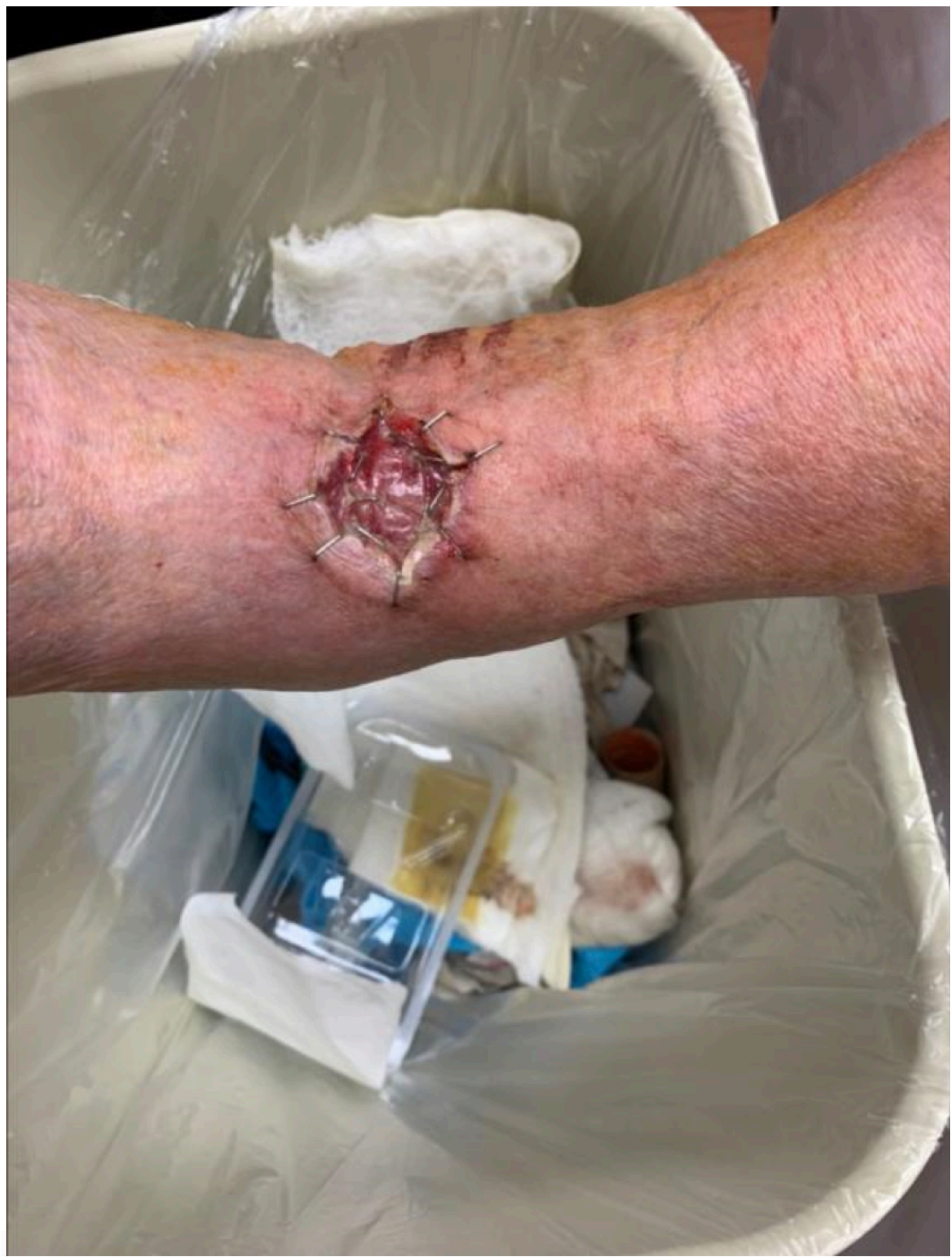# 801 Biodegradable Polyurethane Matrix (BPM) in Small Scale Reconstruction of Malignant Resections in Functional Areas

**DOI:** 10.1093/jbcr/iraf019.332

**Published:** 2025-04-01

**Authors:** Adel Aziz, Maysa Shemmiyeva, Alan Pang, Namratha Mohan

**Affiliations:** Texas Tech University; Texas Tech University Health Sciences Center School of Medicine; Texas Tech University Health Sciences Center School of Medicine; Texas Tech University Health Sciences Center School of Medicine

## Abstract

**Introduction:**

Biodegradable Polyurethane Matrix (BPM) are temporary, biocompatible scaffolds that support tissue regeneration in surgical defects while reducing the need for permanent solutions. Spiradenomas, benign skin tumors from sweat gland cells, often require surgical removal, which can lead to scarring, particularly around joints like the ankle. Preliminary studies show BPMs may be effective in managing defects from malignant resections in critical areas, but their role in resection margins is still an emerging area needing further investigation.

**Methods:**

This case report presents the successful application of a BPM in the reconstruction of a soft tissue defect following wide local excision of a spiradenoma on the right ankle of a 64-year-old male patient, demonstrating the value of these matrices as an essential reconstructive tool in the management of soft tissue defects following malignant resections.

**Results:**

Follow up evaluations showed good graft take of the split-thickness skin graft with good interval healing and smooth contour restoration. Importantly, the patient maintained full range of motion in the right ankle joint, thus highlighting the successful preservation of function in this critical area. Some minor hypergranulation tissue was observed and subsequently treated with silver nitrate. The use of BPM provided reliable and robust soft tissue coverage with optimal functional and aesthetic outcomes and minimal donor site morbidity when compared to skin grafting outcomes alone.

**Conclusions:**

Overall, the use of BPM in this case of spiradenoma excision and subsequent wide local resection in a functional area of the ankle provided robust soft tissue coverage while enabling excellent functional recovery and cosmetic outcomes. The minimal donor site morbidity associated with this approach also further highlights its advantages over traditional skin grafting techniques. The successful application of BPMs in functional areas illustrates their value as an essential reconstructive in the management of soft tissue defects following potentially malignant/malignant resections.

**Applicability of Research to Practice:**

BPMs provide an effective temporary solution for wound healing, as demonstrated in a case where they facilitated skin grafting after wide excision. This approach led to successful graft integration and excellent functional recovery, allowing the patient full ankle mobility. Their ability to preserve function during healing is crucial in joint areas, minimizing complications and enhancing quality of life. Additionally, BPMs reduce donor site morbidity, leading to less discomfort compared to larger graft sites while promoting better healing and mobility outcomes.

**Funding for the Study:**

N/A